# Focal Cortical Lesions Induce Bidirectional Changes in the Excitability of Fast Spiking and Non Fast Spiking Cortical Interneurons

**DOI:** 10.1371/journal.pone.0111105

**Published:** 2014-10-27

**Authors:** Barbara Imbrosci, Angela Neitz, Thomas Mittmann

**Affiliations:** Institute of Physiology, University Medical Center of the Johannes-Gutenberg University Mainz, Mainz, Germany; Georgia State University, United States of America

## Abstract

A physiological brain function requires neuronal networks to operate within a well-defined range of activity. Indeed, alterations in neuronal excitability have been associated with several pathological conditions, ranging from epilepsy to neuropsychiatric disorders. Changes in inhibitory transmission are known to play a key role in the development of hyperexcitability. However it is largely unknown whether specific interneuronal subpopulations contribute differentially to such pathological condition. In the present study we investigated functional alterations of inhibitory interneurons embedded in a hyperexcitable cortical circuit at the border of chronically induced focal lesions in mouse visual cortex. Interestingly, we found opposite alterations in the excitability of non fast-spiking (Non Fs) and fast-spiking (Fs) interneurons in acute cortical slices from injured animals. Non Fs interneurons displayed a depolarized membrane potential and a higher frequency of spontaneous excitatory postsynaptic currents (sEPSCs). In contrast, Fs interneurons showed a reduced sEPSCs amplitude. The observed downscaling of excitatory synapses targeting Fs interneurons may prevent the recruitment of this specific population of interneurons to the hyperexcitable network. This mechanism is likely to seriously affect neuronal network function and to exacerbate hyperexcitability but it may be important to protect this particular vulnerable population of GABAegic neurons from excitotoxicity.

## Introduction

Focal cortical injuries are often followed by perturbations in the excitability of the surviving neuronal networks. It has been assumed that the development of this abnormal neuronal activity is the result of an imbalance between excitation and inhibition, where changes in inhibition are believed to play the most important role [1]. An impaired inhibitory transmission has been reported in several animal models of both traumatic and ischemic brain injury [2], [3]. Functionally, the impaired GABAergic transmission has been attributed to a reduced release of GABA from presynaptic terminals [4], to changes in the expression of GABA receptors [5] and to a dysfunction in Cl^−^ transport [6]. Recent evidence also suggested changes in tonic inhibition, with potential deleterious consequences on neuronal network function and plasticity [7], [8]. A significant reduction in the axonal length and in the number of synaptic contacts formed by fast-spiking interneurons has also been reported following brain lesions [9]. Despite this wealth of knowledge, most reports primarily focused on the output synapses of GABAergic neurons. Therefore, studying the impact of the loss of a cortical area on the excitability of GABAergic cells remains the missing piece of the puzzle for a comprehensive understanding of the cellular mechanisms underlying cortical network dysfunction and hyperexcitability post-injury. In the present study we employed an ex vivo-in vitro model of laser-lesions in mouse visual cortex to study the functional properties of GABAergic interneurons in the cortical network adjacent to the lesion. The injured cortex showed clear signs of hyperexcitability in the first week post-lesion as reveled by a robust increase in the spontaneous neuronal firing from multi-electrode array (MEA) extracellular recordings. Interestingly, we disclosed that the modulation in the excitability of GABAergic cells was cell type-specific. In particular, we observed an elevated excitability in a large subpopulation of GABAergic cells that we identified, based on specific firing properties, as non fast-spiking (Non Fs) interneurons. In contrast, fast-spiking (Fs), parvalbumin-expressing interneurons displayed a reduction in the strength of their excitatory drive and therefore tended to be hypoexcitable. We hypothesize that the specific functional alterations observed at Fs interneurons may represent a mechanism of synaptic downscaling engaged to protect this particular vulnerable population of GABAegic cells from excitotoxicity.

## Materials and Methods

### Ethical statement

This study was carried out in strict accordance with the European Communities Council Directive of September 22nd. 2010 (2010 / 63 / EEC) for care of laboratory animals and after approval of the experimental protocol through the local government ethics committee (Landesuntersuchungsamt Koblenz, G11-1-016). The number of animals was kept to a minimum and all efforts were made to minimize the suffering of the mice. Heterozygous GAD67-GFP knock-in mice were initially generated by Y. Yanagawa [10].

### Cortical lesion induction and electrophysiology

C57BL/6 wild type (n = 12) or GAD67-GFP heterozygous mice (n = 34) at the age of 21 days were anaesthetized by an intraperitoneal injection of a mixture of Ketamine (100 mg/kg) and Xylazine (8 mg/kg). Lesion induction was performed as previously described in [8]. Briefly, a small craniotomy was performed to expose the brain area of interest. The lesion was induced through the open skull under visual control with a 810 nm infrared diode laser (2 Watt) (OcuLight SLx, Iris Medical, USA) in the medial part of the right visual cortex. Age-matched littermates were used as sham-operated controls. After a survival time of 3–5 days the animals were deeply anaesthetized with isoflurane and decapitated. Coronal slices containing the visual cortex (300 µm) were prepared by use of a vibratome (LEICA, VT-1000-S, Germany). The tissue was incubated at room temperature for 1 hour in a standard Artificial CerebroSpinal Fluid (ACSF) containing (in mM): 125 NaCl, 25 NaHCO_3_, 2.5 KCl, 1.5 MgCl_2_, 2 CaCl_2_, 1.25 NaH_2_PO_4_, and 25 D-glucose (pH 7.4) and bubbled with 95% O_2_ and 5% CO_2_. The temperature of the ACSF was kept constant at 33±1°C during all recordings.

#### MEA recordings

**Figure 1:**
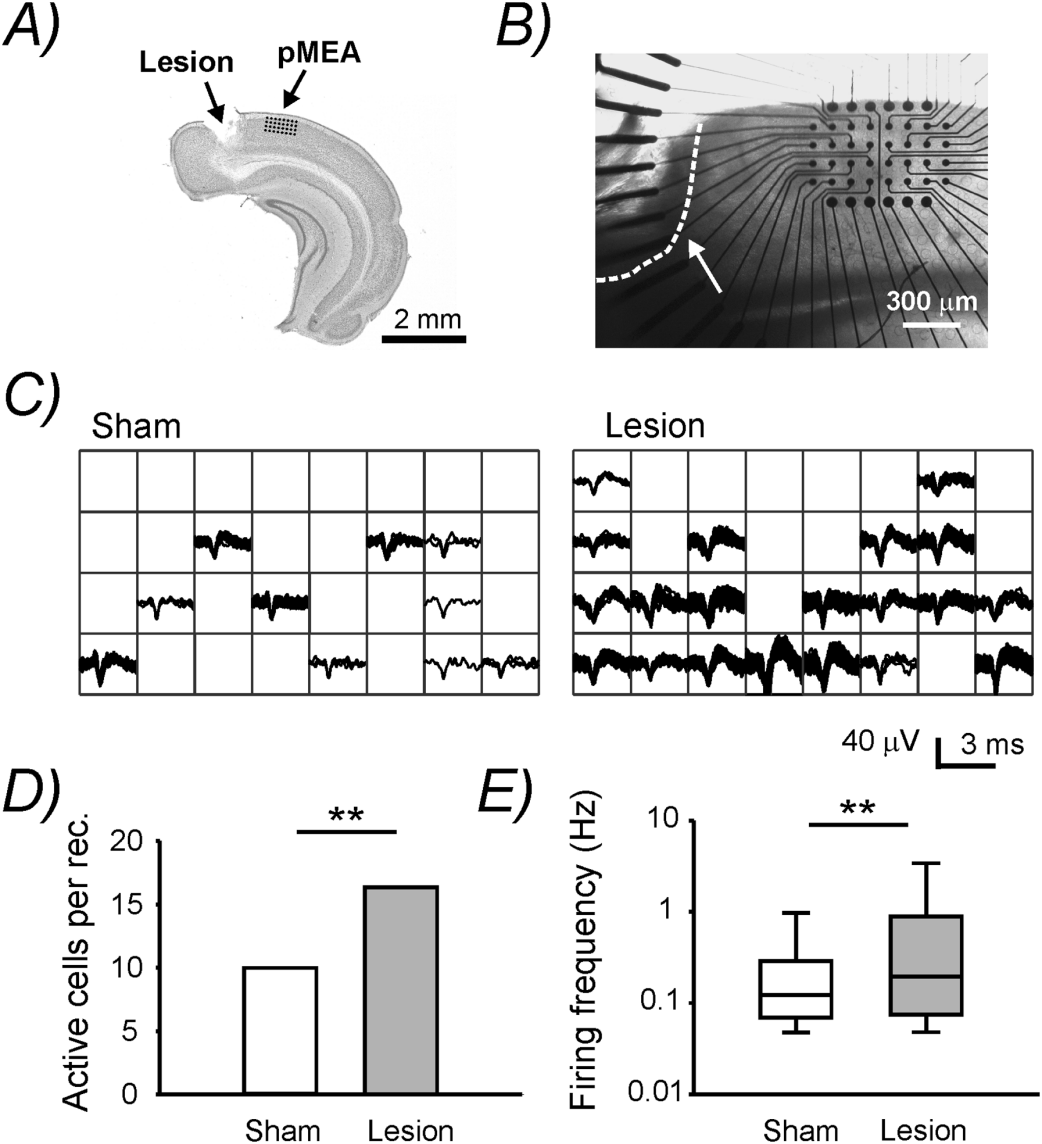
Extracellular pMEA recordings showing an increased spontaneous neuronal activity post-lesion. **A)** Nissl stained coronal section of the right cortical hemisphere containing the lesion in the visual cortex. The drawing illustrates the position of the 32 pMEA recording electrodes on the superficial layers at 1 mm distance from the border of the lesion. **B)** Microphotograph showing the pMEA placed on a injured slice. The white arrow indicates the border of the lesion delimited by the dashed white line. **C)** Peak-aligned detected spikes recorded from the 32 channel pMEA chip during a period of 2 minutes. **D)** Number of active neurons per recording. **E)** Box plot showing the spontaneous neuronal firing rate. The line within the boxes marks the median, the lower and upper boundaries of the boxes indicate the 25th and 75th percentiles and the whiskers below and above the boxes indicate the 10th and 90th percentiles, respectively.

Extracellular recordings of spontaneous firing activity in brain slices were performed with a perforated multi-electrode Array (pMEA) chip (Multi channel Systems, Reutlingen, Germany) equipped with 32 recording and 12 stimulating Titanium nitride (TiN) electrodes. The recording electrodes had a diameter of 30 µm, an impedance ranging from 30 to 50 kΩ and were placed in a 8×4 grid. The inter-electrode distance (centre to centre) was 100 µm. The pMEA chip was mounted beneath a small circular recording chamber. Coronal slices were carefully positioned on the surface of the chip to align the first row of recording electrodes to the upper border of cortical layer 2. In this way the majority of electrodes were located in supragranular layers. The centre of the pMEA was placed at around 1 mm distance from the border of the lesion (**Fig. 1A**). Homotopic regions were selected in sham-operated animals. To improve the contact between slices and electrodes we applied a negative pressure (32–36 mbar) through the perforation. To induce spontaneous activity we perfused the slices on the pMEA with a modified ACSF containing: (in mM): 126 NaCl, 25 NaHCO_3_, 4.3 KCl, 1 MgCl_2_, 1 CaCl_2_, 1.25 NaH_2_PO_4_, and 25 D-glucose bubbled with 95% O_2_ and 5% CO_2_ and supplemented this bathing solution with the muscarinic acetylcholine receptor agonist carbachol (30 µM) and a very low concentration of kainate (500 nM). These pharmacological agents should mimic the higher level of glutamate and the ascending cholinergic inputs normally present *in* *vivo*. Similar concentration of carbachol and kainate have already been used to activate the local network and to induce gamma oscillations in cortical slices [11]. Raw signals, sampled at 25 kHz, were recorded with a MEA2100-acquisition system (Multichannel System, Reutlingen, Germany). MC_Rack software (Multicannel System, Reutlingen, Germany) was used for data acquisition and off-line analysis. To optimize spike detection and sorting the signal was high-pass filtered at 300 Hz. Threshold for spike detection was set to 5X the standard deviation of the noise level. An off-line spike-cluster analysis was used to isolate action potentials from single neurons. The variability in spike amplitude and waveform was used as criteria for spike sorting. The spontaneous firing rate was calculated as the median of spontaneously occurring action potentials during a recording period of 5 min.

#### Patch-clamp recordings

Single slices from GAD67-GFP mice were transferred into a submerged recording chamber superfused (perfusion rate: 3.5 ml/min) with an ACSF containing (in mM): 126 NaCl, 25 NaHCO_3_, 3.5 KCl, 1 MgCl_2_, 1 CaCl_2_, 1.25 NaH_2_PO_4_, and 25 D-glucose bubbled with 95% O_2_ and 5% CO_2_. Whole-cell patch-clamp recordings were performed 1 mm laterally from the border of the lesion and in a homotopic cortical area in sham-operated animals from GFP labeled layers 2/3 interneurons. The intracellular solution contained (in mM): 140 K-gluconate, 8 KCl, 2 MgCl_2_, 4 Na2-ATP, 0.3 Na2-GTP, 10 Na-phosphocreatin, 10 HEPES and 0.5% biocytin. The pH was set to 7.3 with KOH. Access resistance was controlled before and after each voltage-clamp recording. Neurons were discarded if this parameter was either higher than 20 MΩ or changed more than 20%. sEPSCs were measured at −60 mV, at or in close proximity to the reversal potential for GABA_A_ receptors. Evoked EPSCs were produced placing a glass stimulating electrode in layer 4 underneath the recorded cell. Input resistance (Ri) and firing properties were studied by applying a series of 1 second lasting square pulses of hyperpolarizing and depolarizing currents through the patch-clamp electrode (at 0.1 Hz). We started with −100 pA and increased the magnitude of the injected current by 50 pA for each step. During current injection the membrane potential (Vm) of neurons was set to −70 mV. An Axopatch-200B amplifier (AXON Instrument, USA) was used to record electrical signals. Data were filtered at 10 kHz and digitized at 20 kHz using a Digidata-1400 system with PClamp 10 software (Molecular Devices, Sunnyvale, CA, USA). PClamp 10.1 software was used for off-line analysis. The adaptation coefficient of action potential firing was calculated by dividing the inter-spike interval (ISI) of the first 10 by the last 10 action potentials evoked by 1 sec lasting subsaturating somatic current injection. Spike half-width was measured as spike duration at half spike amplitude. Spontaneous excitatory postsynaptic currents (sEPSCs) were semi-automatically identified with Mini Analysis Software (Synaptosoft, USA) and further validated by careful visual inspection. Frequency and amplitude of sEPSCs were calculated in each cell as the median of spontaneous events occurring in a period of 60 sec. To calculate the paired-pulse ratio (PPR) of evoked EPSCs we averaged six to eight consecutive responses acquired every 15 sec. PPR was computed as the ratio between the amplitude of the second to the first EPSC elicited at different inter-stimulus intervals (ISIs). The software Matlab (Mathworks, Natick, MA, USA) and SigmaPlot 2001 (Systat Software, San Jose, USA) were used for graphical visualization of the data.

### Immunohistochemistry

Triple immunofluorescence stainings of biocytin-filled neurons were performed to verify that recordings were obtained from GFP-labeled neurons and to examine parvalbumin expression. Slices were fixed overnight with 4% paraformaldehyde, rinsed with PBS and treated for 90 min with PBS containing 10% normal goat serum, 0.2% Triton X-100, 20% avidin (block A, blocking kit, Vector, USA). Subsequently slices were incubated overnight with the primary antibody rabbit anti-parvalbumin (1∶1000, Swant, Switzerland) diluted in PBS containing 1% normal goat serum, 0.2% Triton X-100, 20% biotin (block B, blocking kit, Vector, USA). Neurons were analyzed on the following day after incubating slices for 90 min with Cy5-conjugated goat anti-rabbit (1∶250, Jackson ImmunoResearch Europe) together with streptavidin-conjugated Cy3 (1∶250, Jackson ImmunoResearch Europe).

### Statistics

Results are presented as mean ± SEM both in the text as well as in table 1. For statistical evaluation of the data we performed an unpaired Student-t test after verifying the normal distribution of the data with one-sample Kolmogorov-Smirnov test. Mann–Whitney U test was applied in case data were not normally distributed.

**Table 1:** Intrinsic membrane and synaptic properties of Fs and Non Fs interneurons in sham-operated and lesion-treated mice.

	Fs Sham	Fs Lesion	Non Fs Sham	Non Fs Lesion
**Spike half-width**	0.345±0.011 ms (10)	0.344±0.015 ms (9)	0.623±0.015 ms (52)	0.639±0.021 ms (48)
**Adapt. Coeff.**	0.774±0.019	0.798±0.042	0.451±0.013	0.477±0.017
**Input resistance**	117.7±12.12 MΩ	109.88±15.35 MΩ	160.98±11.65 MΩ	152.44±10.27 MΩ
**Max. firing rate**	315±23.59 Hz	301±21.9 Hz	102.08±8.8 Hz	97.5±9.13 Hz
**Resting Vm**	−67.20±1.99 mV	−69.22±1.99 mV	−70.13±0.98 mV	−66.12±0.90 mV
**sEPSC amplitude**	22.39±1.80 pA (10)	17.34±1.12 pA (9)	13.93±0.88 pA (33)	13.36±0.54 pA (34)
**sEPSC frequency**	18.07±2.18 Hz	17.82±1.72 Hz	5.07±0.44 Hz	7.99±0.73 Hz
**sEPSC rise time**	0.434±0.024 ms	0.437±0.043 ms	2.521±0.35 ms	0.638±0.041 ms
**sEPSC decay time**	2.889±0.134 ms	2.521±0.35 ms	3.802±0.263 ms	4.153±0.337 ms
**ECA/s**	6.61±1.09 nA/s	5.02±0.61 nA/s	5.96±0.64 nA/s	9.05±0.96 nA/s
**PPR (20** **ms)**	-	-	1.49±0.14 (9)	1.20±0.07 (13)
**PPR (30** **ms)**	-	-	1.32±0.06 (11)	1.42±0.12 (14)
**PPR (50** **ms)**	-	-	1.28±0.12 (10)	1.29±0.11 (14)

The numbers in parenthesis indicate the number of recorded neurons.

## Results

### Hyperexcitability in the visual cortex post-lesion

**Figure 2:**
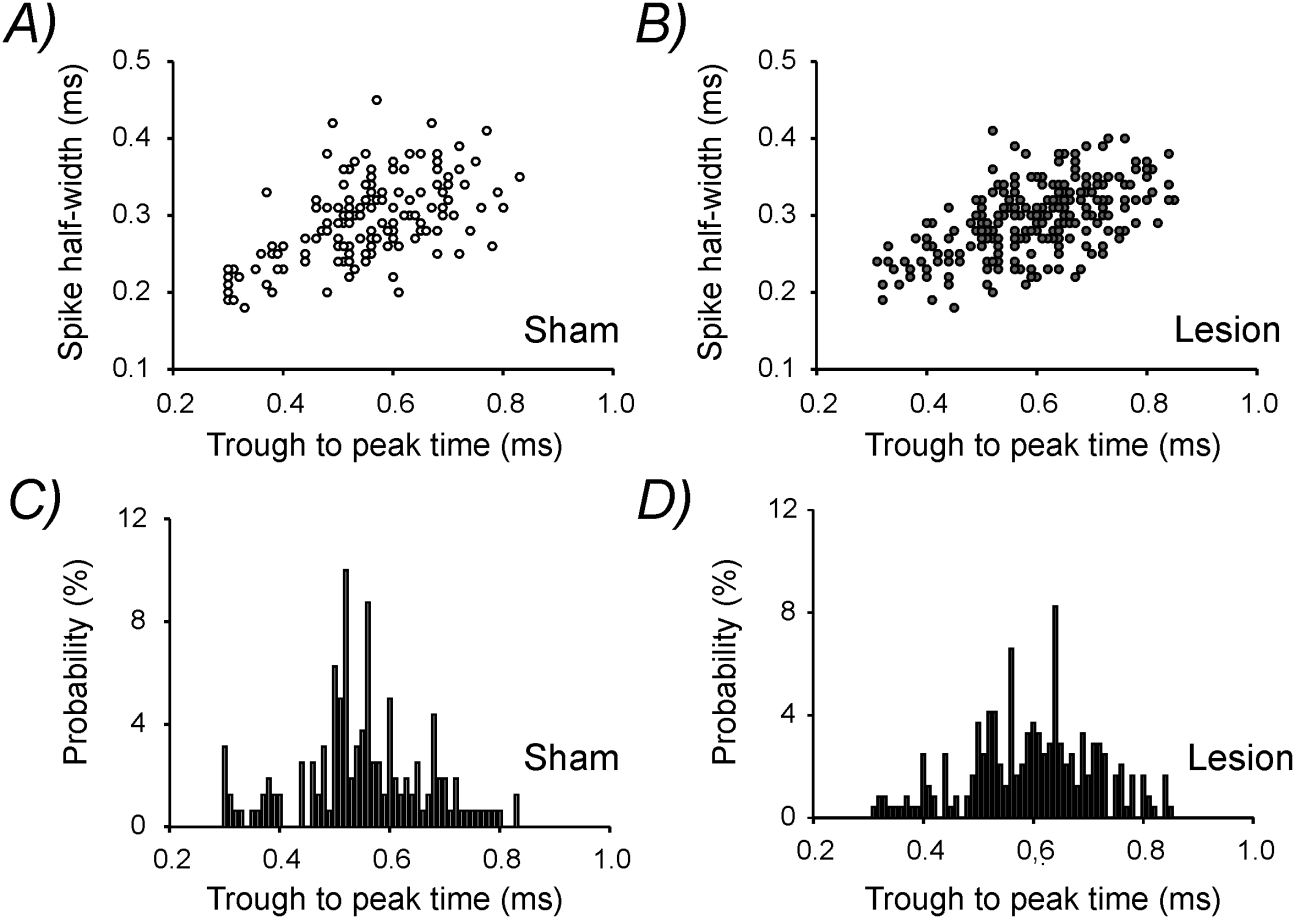
Analysis of the extracellular spike waveforms. Plot of spike half-width versus trough-to-peak time in **A)** sham-operated and **B)** lesion-treated animals for each detected active neuron. K-mean cluster analysis performed on these two sets of data failed to produce two clusters which could be representative of pEX and pIN cells (not shown). Histogram distribution of trough-to-peak time for **C)** sham-operated and **D)** lesion-treated animals.

Previous studies often reported highly excitable cortical networks surrounding a brain injury. Thus, we firstly measured the activity of the surviving cortical microcircuits in our laser-lesion model. We analyzed the occurrence of spontaneous action potentials in acute cortical slices from sham-operated and lesion-treated animals. We simultaneously recorded from 32 electrodes with a pMEA placed at the border of the lesion and in the homotopic area in control mice (**Fig. 1A, B**). We found that the number of spontaneously active cells detected in each recording (sham: 10±1.10, n slice = 16 from 6 mice; lesion 16.33±1.50 n slice = 15 from 6 mice; p<0.005; **Fig. 1C–D**) as well as the firing frequency of the spontaneously active neurons (sham: 0.122±0.121 Hz n = 160 from 6 mice; lesion: 0.196±0.144 Hz, n = 244 from 6 mice; p<0.005; **Fig. 1C, E**) were significantly higher in the lesion group. These findings strongly suggest that the cortical tissue at 1 mm distance from the border of the lesion is characterized by a hyperexcitable neuronal network. Extracellular *in* *vivo* recordings allow to distinguish between putative excitatory (pEX) and fast-spiking inhibitory (pIN) neurons based on the extracellular signature of the spikes. We also attempted to distinguish pEX and pIN cells in our dataset to disclose a potential differential effect of the lesion on these two neuronal populations. In vivo, the trough-to-peak time of the spike waveform has proven to be the best parameter to separate pEX from pIN cells [12]. Generally, the great majority of fast-spiking interneurons presents a very short trough-to-peak time (generally below <0.45 ms) while the majority of excitatory neurons displays values larger than 0.45–0.50 ms [13], [14]. When we plotted the trough-to-peak time against the spike half-width for each detected active neuron (**Fig. 2A, B**) we could not identify two separate clusters at or close to these trough-to-peak time values by k-means cluster analysis. This suggests that under in vitro experimental conditions it may be more complex to distinguish between pEX and pIN neurons compared to in vivo data. In addition, in our study, this type of analysis was further complicated since we disclosed a significant prolongation in the mean trough-to-peak time following lesion (sham: 551.3±9.05 µs; lesion: 597.6±7.46 µs; p<0.001). This could also be observed as a rightward shift in the histogram distribution of the trough-to-peak time (**Fig. 2C, D**) and may reflect a lesion-induced increase in the duration of action potential repolarization.

### Identification of fast-spiking and non fast-spiking interneurons

**Figure 3:**
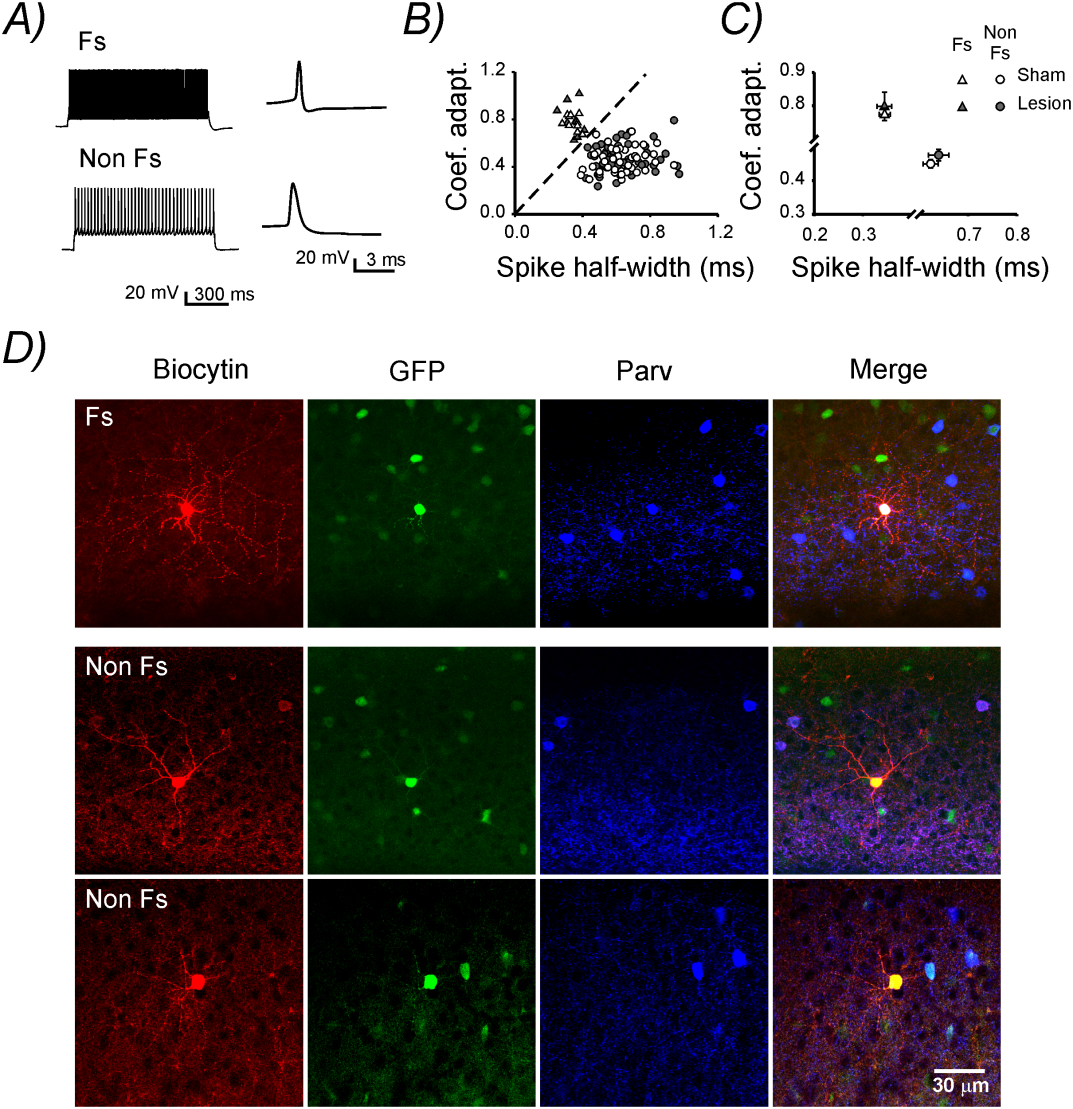
Characterization of fast spiking (Fs) and Non fast spiking (Non Fs) interneurons. **A)** Voltage traces in response to 1 sec-lasting, supra-threshold somatic current injection (100 pA) in a representative Fs (top) and Non Fs (bottom) interneuron. The inset on the right emphasizes the different action potential waveform in the two cell types. **B)** Plot displaying the coefficient of adaptation versus the spike half-width in each recorded cell. Note the presence of two non overlapping clusters of neurons separated by the dashed line. **C)** Mean coefficient of adaptiation and spike half-width in each experimental group. **D)** Triple immunofluorescence stainings for biocytin (red), GFP (green) and Parvalbumin (blue) in one representative Fs (top) and two Non Fs (bottom) neurons.

To further investigate potential functional alterations in inhibitory interneurons embedded into this hyperexcitable neuronal network we performed patch-clamp recordings from layer 2/3 GABAergic interneurons in heterozygous GAD67–GFP knock-in mice. In this mouse line all different GABAergic interneuron subtypes are labeled with GFP [10]. Post-hoc identification, obtained in a subset of biocytin-filled cells, confirmed that the recorded neurons were always positive for GFP (29/29, **Fig. 3D**). To distinguish Fs interneurons from other interneuron subclasses we plotted the adaptation coefficient versus the spike half-width in each recorded neuron from both sham-operated and lesion-treated animals. This method allowed the identification of two separately clusters of cells. One cluster was populated by Fs interneurons, characterized by very little spike frequency adaptation (aptation coefficient >0.6) and narrow action potentials. The second cluster contained neurons with a significantly lower adaptation coefficient (p<0.001) and wider action potentials (p<0.001; **Fig. 3A–C**). This second heterogeneous population of interneurons will be referred to as Non Fs. Previously, a similar approach was employed to separate Fs from somatostatin-expressing neurons [15]. Here, we adapted this method to isolate Fs neurons from the remaining subtypes of GABAergic interneurons. To further explore the identity of Fs and Non Fs interneurons, we performed post-hoc morphological and immunohistochemical analysis on a subset of the recorded cells. Consistent with previous reports, Fs interneurons identified in our recordings always displayed a multipolar morphology and expressed the calcium-binding protein, parvalbumin (8 of 8 cells). This suggested that they were presumably perisomatic innervating basket cells. Conversely, Non Fs interneurons presented variable morphology (multipolar, bipolar, etc) and did not express parvalbumin (0 or 21 cells) suggesting that they represent a broad spectrum of interneuron subtypes (**Fig. 3D**).

### Lesion-induced changes in resting membrane potential in Non Fs interneurons

**Figure 4:**
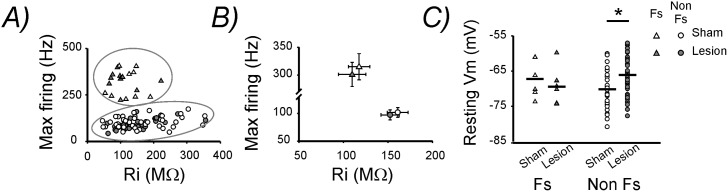
Intrinsic properties of interneurons post-lesion. **A)** Plot showing the maximal firing rate (max. firing) versus the input resistance (Ri) in each recorded cell. The max. firing was measured in response to a saturating, one sec-lasting, somatic current injection. **B)** Mean max. firing and Ri in each experimental group. **C)** Plot showing the resting membrane potential (resting Vm) in each recorded cell. The black horizontal lines represent the mean resting Vm for each experimental group.

Next we studied the intrinsic membrane properties of the two identified subgroups of GABAergic cells post-lesion. The mean spike half-width and adaptation coefficient were neither altered in Fs nor in Non Fs interneurons (**Table 1** **,** **Fig. 3C**). Subsequently, we analyzed the input resistance (Ri) and maximal (max.) firing rate (upon somatic current injection) of each recorded neuron. In accordance with previous studies, Fs neurons displayed a low Ri and high max. firing rate [16], [17]. This results in a clear cell-cluster separation when plotting max. firing rate versus Ri (**Fig. 4A**), further corroborating the validity of our classification criteria (**Fig. 3B**). The lesion caused no alteration in either Ri or max. firing rate in both Fs and Non Fs neurons (**Table 1** **,** **Fig. 4B**). In contrast the resting membrane potential (resting Vm) was significantly depolarized in Non Fs neurons but remained unaltered in Fs neurons (**Table 1** **,** **Fig. 4C**).

### Lesion-induced changes in excitatory inputs onto Fs and Non Fs neurons

**Figure 5:**
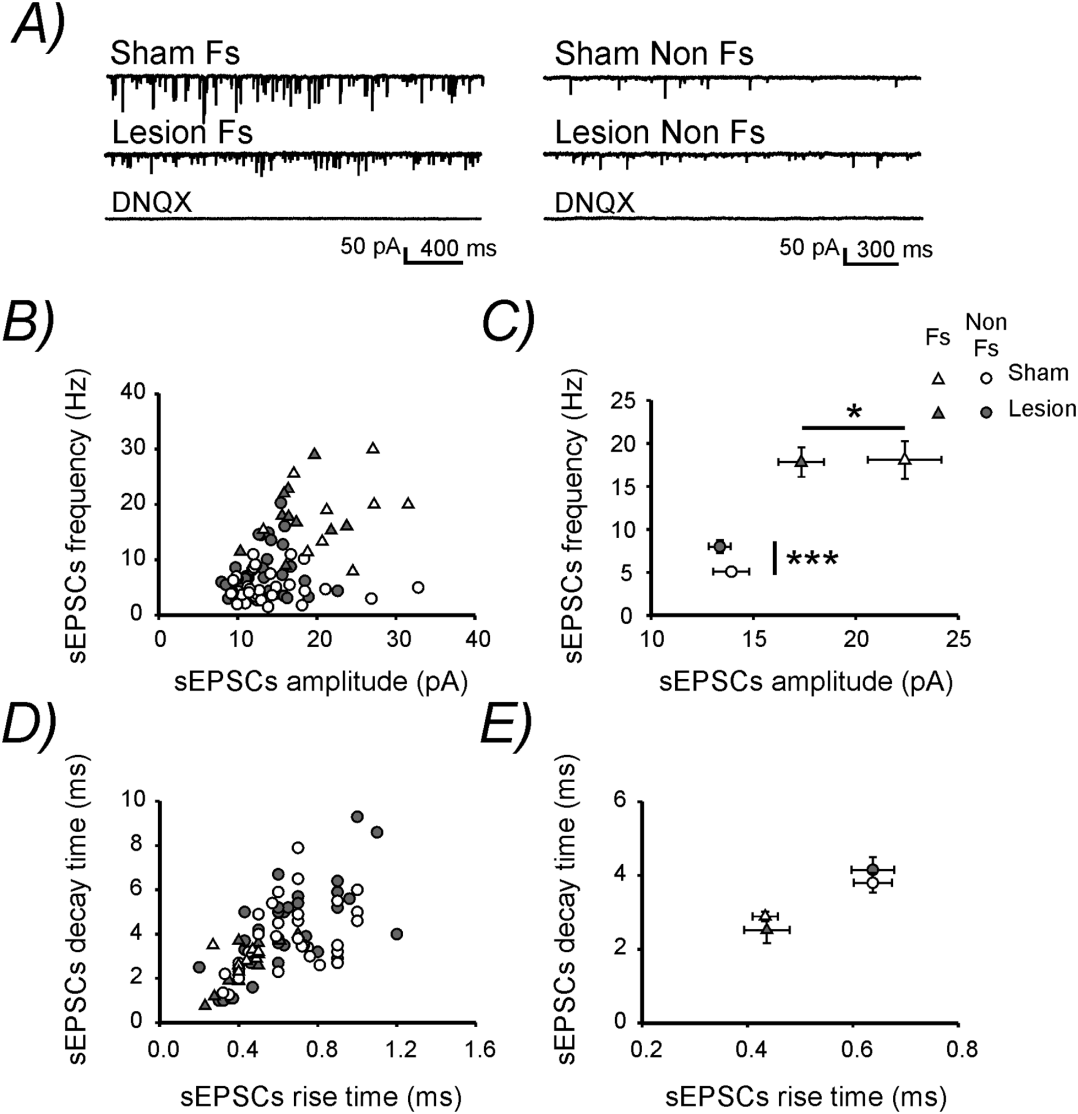
Lesion-induced changes in spontaneous excitatory synaptic inputs onto interneurons. **A)** Representative sEPSCs traces recorded at −60 mV in Fs (left) and Non Fs (right) layers 2/3 interneurons. The application of DNQX (20 µM) abolished all signals confirming that they were due to the activation of AMPA receptors. **B)** Plot showing sEPSCs frequency versus sEPSCs amplitude in each recorded neuron. **C)** Mean sEPSCs frequency and amplitude in each experimental group. **D)** Plot showing sEPSCs decay time versus sEPSCs rise time in each recorded neuron. **E)** Mean sEPSCs decay and rise time in each experimental group.

**Figure 6:**
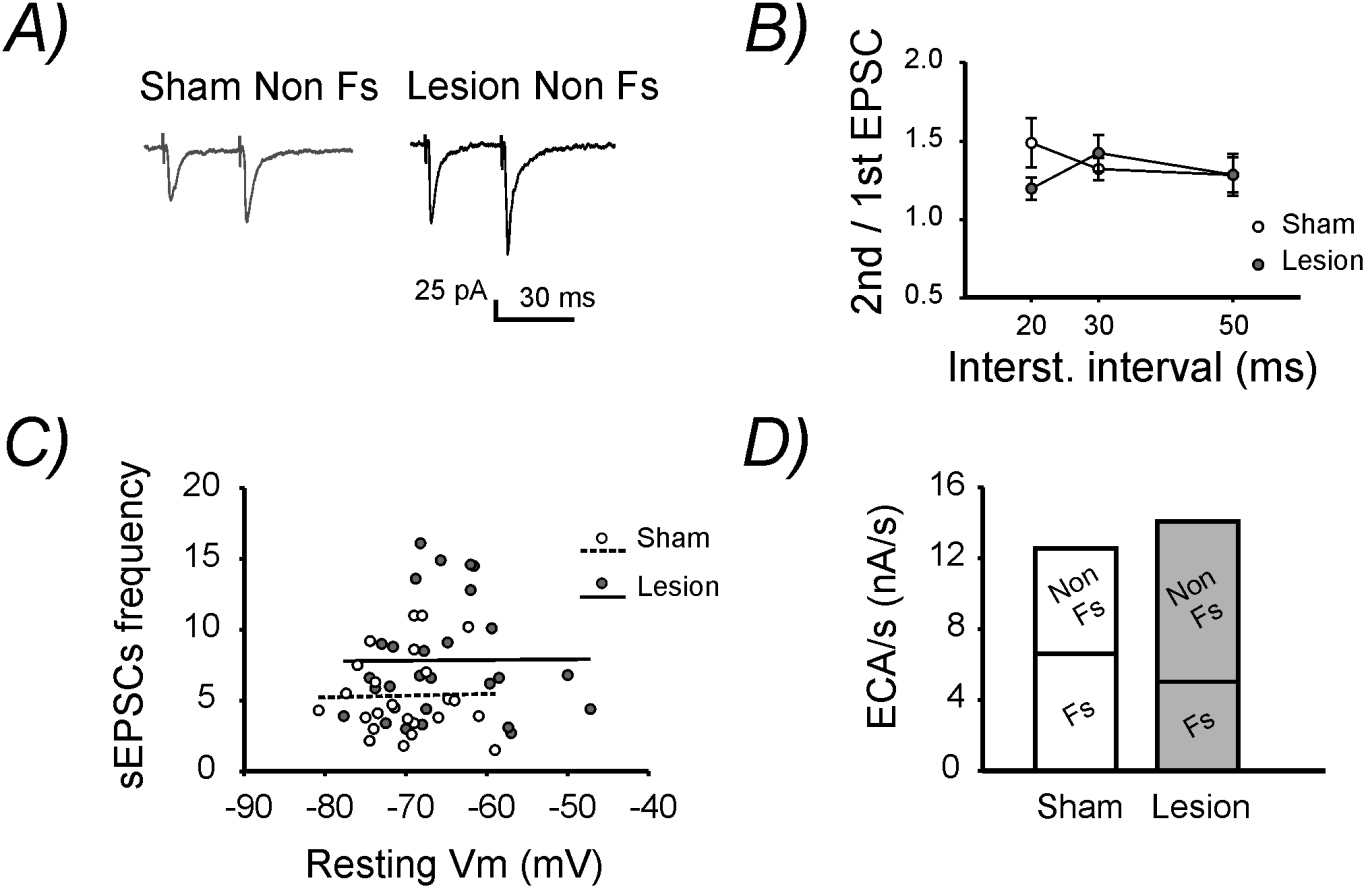
Changes in excitatory synaptic inputs onto Fs and Non Fs interneurons. **A)** Representative EPSCs recorded at −60 mV evoked by pairs of synaptic stimulations with an interstimulus interval (ISI) of 30 ms recorded from two Non Fs interneurons (in sham-operated and lesion animals). **B)** Summary diagram of the mean paired-pulse ratio (PPR) for different ISIs. **C)** Plot showing sEPSCs frequency versus resting membrane potential (Vm) in Non Fs interneurons showing no correlations between the two parameters. **D)** Excitatory current amplitude per second (ECA/s) experience by a theoretical population of Fs and Non Fs interneurons.

Next, we measured sEPSCs in neurons voltage clamped at −60mV. In line with previous studies [15], [17] Fs neurons had a significantly higher sEPSCs frequency, amplitude and a significantly faster rise and decay time as compared to Non Fs cells (**Fig. 5A–E**). Interestingly, the lesion led to differential effects in the two subpopulations of interneurons. The amplitude of sEPSCs was reduced selectively in Fs neurons. On contrary, only Non Fs neurons showed a higher sEPSCs frequency post-lesion (p = 0.001), (**Fig. 5B, C**). Rise-time and decay-time constants of sEPSCs in both Fs and Non Fs cells remained unaltered (**Table 1** **,** **Fig. 5D, E**). Alterations in the frequency of sEPSCs are often attributed to changes in neurotransmitter release. We tested this by analyzing excitatory postsynaptic currents induced by extracellular presynaptic stimulation. These experiments were conducted on Non Fs cells only, since changes in sEPSCs frequency were exclusively observed in these interneurons. The PPR calculated from two consecutive EPSCs evoked at different ISIs was not altered by the lesion (**Table 1** **,** **Fig. 6A, B**). Since alterations in PPR are often interpreted as changes in the release probability of neurotransmitter in pyramidal neurons [18], our results suggest an unchanged glutamate release at these synapses.

Independent on the mechanisms, the increased glutamatergic transmission onto Non Fs interneurons may also influence their membrane properties. In this regard, it has been reported that excessive glutamate can lead to sustained membrane depolarization [19] and ultimately to excitotoxicity [20]. Therefore, we asked whether the increased occurrence of sEPSCs in Non Fs cells could be responsible for the depolarized resting Vm specifically observed in this interneuronal population. Arguing against this hypothesis we found no correlation between the frequency of sEPSCs and the resting Vm in Non Fs neurons (sham: r = 0.023, lesion: r = 0.008), (**Fig. 6C**).

Finally, to evaluate whether the bidirectional effects of the lesion on Fs (reduced sEPSCs amplitude) and Non Fs (increased sESPCs frequency) neurons were equally strong, or whether one effect prevailed over the other we estimated, based on our experimental data, the overall excitatory drive experienced by a theoretical population of GABAergic interneurons. First, we multiplied the sEPSCs amplitude by the sEPSCs frequency in each neuron and we calculated the mean value for both Fs and Non Fs interneurons. Since the sEPSCs frequency is expressed in *Hz* this value represents the sum of the amplitude of all the sEPSCs occurring, on average, in one second in Fs and Non Fs interneurons. We named the obtained parameter “excitatory current amplitude experienced in one second” (ECA/s). Importantly, our experimental data show that the contribution of Fs and Non Fs interneurons is not equal. In fact, interneurons classified as Fs constitute only 16% (19 of 119 cells) of the all recorded cells while Non Fs cells constitute the remaining 84% (100 of 119 cells). To take into account the different proportion of these two neuronal populations we multiplied the ECA/s of Fs and Non Fs neurons by 16 and 84, respectively. As expected the ECA/s in sham-operated animals was slightly higher than lesion-treated mice in Fs interneurons but significantly lower in Non Fs interneurons (p<0.05), (**Table 1** **,** **Fig. 6D**). As a result, the sum of these two values, which should theoretically represent the ECA/s experience by a population of 100 interneurons, was slightly higher in lesion-treated mice (sham: 12,56 nA/s; lesion: 14,07 nA/s), (**Fig. 6D**). This result suggests that at the level of neuronal network the reduced sEPSCs amplitude in Fs interneurons is likely to compensate partially, but not completely, for the increased excitability in Non Fs interneurons following lesion.

## Discussion

Hyperexcitability in neuronal networks is a pathological condition which often lead to neurological deficits and to the generation of epileptic seizures [21]. One common cause of hyperexcitability is a localized brain damage. Following focal cortical injuries, an abnormal neuronal activity is often found in the adjacent, surviving cortical structures [5], [22]. The time window and the spatial extent of lesion-induced changes in neuronal excitability can vary depending on lesion size, etiology and affected brain areas. Nonetheless a typical spatio-temporal distribution could be recognized in different models of cortical lesion. At a very early stage, primarily in the first two days post-lesion, the close vicinity of the injury is often functionally suppressed [23], [24]. On contrary the cortical area surrounding this rim of reduced activity is frequently hyperactive. Hyperexcitability post-lesion has been described as an increase in the spontaneous as well as in the evoked firing of principal neurons both in vivo [22] and in vitro [25], [26]. Generally, it reaches a peak in the first few days after injury [5], [22] and spreads relatively far (in rodents up to 4 mm) from the border of the lesion [5], [23], [25], occasionally even into remote brain areas [5], [26].

It is believed that this pathophysiological process is caused to a considerable extent by a weakening in GABAergic neurotransmission [1]–[4], [8]. In support of this hypothesis changes in inhibition have been reported to occur in a temporal window similar to the changes in neuronal excitability. Generally a lesion-induced suppression of inhibition can already be observed one day following lesion induction, it reaches a peak in the first week, and then, it slowly (sometimes only partially) recovers in the course of the following one, two months after the lesion induction [1]. Despite the well documented dysfunction of the cortical inhibitory system it is largely unknown, whether a cortical lesion does also trigger alterations in the intrinsic membrane properties and synaptic inputs of inhibitory interneurons themselves. The present study was therefore designed to explore the functional behavior of GABAergic interneurons embedded into an abnormally excitable cortical microcircuit surrounding a localized, infrared laser-induced cortical damage. In a previous study from our laboratory robust changes in inhibitory transmission were found 1 mm laterally from the lesion [8]. At further distances of 2 to 3 mm the effects of the lesion on inhibition were still present but to a minor extent (our unpublished observation). Based on these findings all recordings in the present study were performed at 1 mm distance from the lesion where the effect on inhibition should be more strongly expressed. Firstly, we performed MEA extracellular recordings to ascertain that the apparently healthy cortical tissue lateral to the lesion was indeed hyperexcitable. As expected, we found an increase in spontaneous firing in superficial cortical layers (**Fig. 1**). As already mentioned, this higher excitatory state may be caused by an impaired synaptic transmission between inhibitory and excitatory cells, by an increased activity of NMDA receptors or by an enhanced glutamatergic transmission among principal neurons [4], [27]. Furthermore, alterations in the intrinsic properties of principal cells may also contribute to the development of hyperexcitability post-injury [25].

After we confirmed that the tissue surrounding the lesion presented an abnormal neuronal activity, we wanted to investigate potential functional alterations in inhibitory interneurons embedded into this hyperexcitable neuronal network. Commonly, in vivo recordings, allow to distinguish between pEX and pIN neurons by extracting some features from the extracellular spike waveforms [12]. However, in contrast to the data obtained in vivo, our in vitro data set did not allow to distinguish between these two different neuronal populations (**Fig. 2**). Therefore, to gain further insight into the effect of lesion on inhibitory interneurons we performed patch-clamp recordings from layer 2/3 inhibitory interneurons in GAD67–GFP knock-in mice [10]. The analysis of the firing properties of the recorded GFP-labeled cells allowed us to distinguish Fs, parvalbumin-expressing interneurons from a large and heterogeneous group of cells that we named Non Fs. Fs interneurons could be clearly discriminated on the basis of their short duration action potentials, relatively low spike frequency adaptation and their high firing frequency (**Fig. 3** **,** **4**). Surprisingly, the lesion caused bidirectional alterations in the excitability of these two neuronal populations. Fs interneurons displayed a reduction in the amplitude of spontaneous excitatory synaptic inputs (**Fig. 5**). In contrast, Non Fs interneurons showed a significantly depolarized resting Vm and a higher sEPSCs frequency (**Fig. 4** **,** **5**).

The increased occurrence of sEPSCs in Non Fs interneurons was not associated by changes in the PPR (**Fig. 6**). These results suggest an unaltered release probability of glutamate at these synapses. Structural alterations leading to an increased excitatory connectivity are also unlikely since they require weeks to develop [28]. Alternatively, the higher sEPSCs frequency might be caused by an increased release of glutamate from glia [29] or by an increase in spontaneous firing of principal neurons which was reported in a previous study from our laboratory [26] and it was further corroborated by our MEA extracellular recordings. Finally, the higher sEPSCs frequency was not correlated with the positive shift in resting Vm suggesting that the membrane potential depolarization observed at Non Fs interneurons cannot be explained by an excessive glutamatergic neurotransmission. Possibly, the depolarizing shift in resting Vm can be attributed to a reduction in the activity of the Na^+^/K^+^ ATPase. In support of this hypothesis, a functional impairment of this electrogenic pump has often been observed following brain injury [30]–[32]. Furthermore, similarly to our findings, in a traumatic brain injury model the depolarizing shift in resting Vm was also reported to be neuron type specific [31].

Taken together our data demonstrate that the cortical circuit surrounding the lesion is characterized by an increased excitability which is visible at the network level, as an increase in spontaneous firing, as well as at the cellular level as an increase in the frequency of sEPSCs at Non Fs interneurons. Furthermore, we demonstrated a lesion differential modulation of Fs and Non Fs interneurons with respect to their glutamatergic synaptic inputs. On the one hand the downscaling of excitatory synapses onto Fs interneurons may shift the excitatory-inhibitory balance in favor of the former and therefore exacerbate hyperexcitability. On the other hand the increased excitability of Non Fs interneurons may increase the level of inhibition if the probability of GABA release at synaptic terminals from this neuronal population is not severely compromised post-lesion [8]. To predict the net effect of these bidirectional alterations on the overall neuronal network excitability we estimated the excitatory synaptic strength experience by a theoretical population of Fs and Non Fs interneurons (see result section for details). Our data suggest that at the level of the neuronal network the downscaling of excitatory synapses onto Fs interneurons may only partially compensate for the increased excitability in Non Fs interneurons following lesion. Therefore, our measurements suggest that the net effect of the lesion may be a slight increase in the excitability of interneurons. Nonetheless, it is important to mention that our method does not take into consideration potential differences in the intrinsic excitability of the two neuronal types. Furthermore, it is also important to bear in mind that the group of Non Fs interneurons comprises a largely heterogeneous population of inhibitory cells. Recent studies suggested that specific subgroups of Non Fs interneurons, such as vasoactive intestinal polypeptide (VIP)- or somatostatin (SOM)-positive interneurons, preferentially target other inhibitory neurons [33], [34]. Therefore, by mainly providing inhibition to other inhibitory cells their increased activity is likely to have a net disinhibitory (instead of inhibitory) effect.

An additional interesting observation is that, the lesion-mediated hyperexcitability, seems to specifically spare Fs interneurons. Mechanistically, the reduction in the amplitude of sEPSCs may prevent the detection of the smallest spontaneous synaptic events observed in this interneuronal population. This may explain why we failed to observe an increased occurrence of sEPSCs in Fs interneurons. The downregulation of the amplitude of sEPSCs in Fs interneurons may represent a homeostatic form of synaptic plasticity acting to stabilize the firing activity of Fs interneurons. In this regard, recent evidence suggests that neurons have the ability to detect alterations in their own firing activity −mainly through calcium-dependent mechanisms − and to finely adjust the strength of their synaptic inputs in order to maintain their firing rate within a physiological range [35]. However, if at the cellular level these changes are prone to stabilize the activity of Fs interneurons they cannot be considered homeostatic at the level of neuronal network. Indeed, in a hyperexcitable cortical network, a reduced excitatory drive onto Fs interneurons may reduce the strength of their inhibitory output and thereby exacerbate hyperexcitability. The reason why these changes take place despite they are likely to move the network away from homeostasis may be found in the singular physiological properties of Fs neurons. Recent studies highlighted the fact that Fs cells are particularly vulnerable to perturbations in the central nervous system and are characterized by a very high metabolism and particular sensitivity to oxidative stress when compared to other neuronal subclasses [36]. Based on this evidence it is tempting to hypothesize that the reduced excitability of Fs interneurons may represent an effort to preserve this highly vulnerable population of cells from excitotoxicity and/or to save energy in a high metabolic demanding pathological context. Furthermore, the physiological function of Fs interneurons is not only needed for a fine regulation of the level of neuronal activity but it is also important to guaranty a proper function of cortical circuits. Fs-perisomatic innervating interneurons are well known for their capacity to precisely regulate the spike timing of principal neurons [37]. Furthermore, coordinating the firing of large neuronal ensembles, Fs cells have also a key role in the generation of high frequency oscillations [38] essential for a number of cognitive functions and learning paradigms [39]–[41]. This evidence suggests that a reduced recruitment of this interneuron subclass may not only promote hyperexcitability but it may also result in a deficit of neuronal information processing and, in case a large cortical area is affected, in cognitive and memory impairments.
